# Supplementary material for: Identification and functional analysis of non-coding regulatory small RNA FenSr3 in Bacillus amyloliquefaciens LPB-18
Source: PeerJ. 2023 May 15;11:e15236. doi: 10.7717/peerj.15236 (PMC10194069; doi:10.7717/peerj.15236)
Supplement: Supplemental Information 4 [file peerj-11-15236-s004.zip › KO/CK-vs-T1_map/map04120.html]

KEGG PATHWAY: Ubiquitin mediated proteolysis - Reference pathway


|  |  |
| --- | --- |
| **Ubiquitin mediated proteolysis - Reference pathway** |  |

[
Pathway menu
| Organism menu
| Pathway entry
| Show description
| User data mapping
]

Protein ubiquitination plays an important role in eukaryotic cellular processes. It mainly functions as a signal for 26S proteasome dependent protein degradation. The addition of ubiquitin to proteins being degraded is performed by a reaction cascade consisting of three enzymes, named E1 (ubiquitin activating enzyme), E2 (ubiquitin conjugating enzyme), and E3 (ubiquitin ligase). Each E3 has specificity to its substrate, or proteins to be targeted by ubiquitination. Many E3s are discovered in eukaryotes and they are classified into four types: HECT type, U-box type, single RING-finger type, and multi-subunit RING-finger type. Multi-subunit RING-finger E3s are exemplified by cullin-Rbx E3s and APC/C. They consist of a RING-finger-containing subunit (RBX1 or RBX2) that functions to bind E2s, a scaffold-like cullin molecule, adaptor proteins, and a target recognizing subunit that binds substrates.

|  |  |  |
| --- | --- | --- |
| Reference pathway | 184% 150% 122% 100% 82% 67% 55% | 图片下载 |
